# Delivery mode for prolonged, obstructed labour resulting in obstetric fistula: a retrospective review of 4396 women in East and Central Africa

**DOI:** 10.1111/1471-0528.16047

**Published:** 2020-01-02

**Authors:** CJ Ngongo, TJIP Raassen, L Lombard, J van Roosmalen, S Weyers, M Temmerman

**Affiliations:** ^1^ RTI International Seattle WA USA; ^2^ Nairobi Kenya; ^3^ Cape Town South Africa; ^4^ Athena Institute VU University Amsterdam Amsterdam the Netherlands; ^5^ Leiden University Medical Centre Leiden the Netherlands; ^6^ Department of Obstetrics and Gynaecology Ghent University Hospital Ghent Belgium; ^7^ Centre of Excellence in Women and Child Health Aga Khan University Nairobi Kenya; ^8^ Faculty of Medicine and Health Science Ghent University Ghent Belgium

**Keywords:** Assisted vaginal delivery, caesarean section, destructive delivery, obstetric fistula, stillbirth, vacuum extraction

## Abstract

**Objective:**

To evaluate the mode of delivery and stillbirth rates over time among women with obstetric fistula.

**Design:**

Retrospective record review.

**Setting:**

Tanzania, Uganda, Kenya, Malawi, Rwanda, Somalia, South Sudan, Zambia and Ethiopia.

**Population:**

A total of 4396 women presenting with obstetric fistulas for repair who delivered previously in facilities between 1990 and 2014.

**Methods:**

Retrospective review of trends and associations between mode of delivery and stillbirth, focusing on caesarean section (CS), assisted vaginal deliveries and spontaneous vaginal deliveries.

**Main outcome measures:**

Mode of delivery, stillbirth.

**Results:**

Out of 4396 women with fistula, 3695 (84.1%) delivered a stillborn baby. Among mothers with fistula giving birth to a stillborn baby, the CS rate (overall 54.8%, 2027/3695) rose from 45% (162/361) in 1990–94 to 64% (331/514) in 2010–14. This increase occurred at the expense of assisted vaginal delivery (overall 18.3%, 676/3695), which declined from 32% (115/361) to 6% (31/514).

**Conclusions:**

In Eastern and Central Africa, CS is increasingly performed on women with obstructed labour whose babies have already died in utero. Contrary to international recommendations, alternatives such as vacuum extraction, forceps and destructive delivery are decreasingly used. Unless uterine rupture is suspected, CS should be avoided in obstructed labour with intrauterine fetal death to avoid complications related to CS scars in subsequent pregnancies. Increasingly, women with obstetric fistula add a history of unnecessary CS to their already grim experiences of prolonged, obstructed labour and stillbirth.

**Tweetable abstract:**

Caesarean section is increasingly performed in African women with stillbirth treated for obstetric fistula.

## Tweetable abstract

Caesarean section is increasingly performed in African women with stillbirth treated for obstetric fistula.

## Introduction

Childbirth involves significant risks for women and newborns. These risks reflect global inequity: 99% of all maternal deaths and stillbirths occur in low‐income countries.^1^, ^2^ Quality intrapartum care can prevent most maternal deaths caused by direct obstetric complications, which has led to a focus on quality basic and comprehensive emergency obstetric care.^3^, ^4^ Caesarean section (CS) saves maternal and newborn lives, especially in countries with low CS rates and high maternal mortality ratios.^5^, ^6^ Many African countries have large, unmet needs for CS.^6^, ^7^, ^8^

Compared with vaginal delivery, however, CS is associated with higher risks of maternal and perinatal death and newborn sepsis, as well as maternal morbidity from abnormal invasive placentation and uterine scar rupture in subsequent pregnancies.^9^, ^10^, ^11^, ^12^ Maternal and perinatal deaths following CS are disproportionately high in sub‐Saharan Africa, where studies have identified 5.4^13^ and 10.9^14^ maternal deaths per 1000 live births, at least 50 times higher than mortality after CS in high‐income countries.^13^ The seriousness of CS complications in resource‐limited settings reinforces the importance of appropriate labour management and restrained CS decision‐making.^11^, ^14^ CS should only be performed when clear benefits are anticipated outweighing the additional risks and higher costs.^15^, ^16^

Alternative approaches can resolve prolonged labour with vaginal delivery. In the first stage, artificial rupture of membranes and oxytocin augmentation can be used to accelerate labour. In the second stage, vacuum extraction or forceps are good options to pursue vaginal delivery. In women with obstructed labour and intrauterine fetal death, destructive delivery is the method of choice if obstruction makes vacuum delivery impossible.^17^

Genito‐urinary and recto‐vaginal fistulas are consequences of women's insufficient access to timely emergency obstetric care in situations where cephalopelvic disproportion, malpresentation, or malposition cause prolonged, obstructed labour. Without intervention, obstruction leads to pressure necrosis and fetal death. Obstetric fistula is a chronic, severe morbidity associated with long‐term physical, emotional, psychological, social and economic consequences.^18^, ^19^ Proper monitoring of labour with timely intervention could prevent obstetric fistula in low‐resource settings, as it already has in well‐resourced settings.^20^

The objective of this paper is to assess trends in modes of delivery and stillbirth rates over time among women with obstructed labour who sought treatment for obstetric fistulas.

## Patients and methods

This retrospective record review evaluated mode of delivery over time among women presenting with fistula‐related incontinence in Tanzania, Uganda, Kenya, Malawi, Rwanda, Somalia, South Sudan, Zambia and Ethiopia (see Supplementary material, Table S1). Women seeking fistula repair were interviewed in 82 facilities, largely district and mission hospitals. They had developed fistula during childbirth in an unknown, larger number of facilities some time before seeking fistula repair. Data were collected between June 1994 and December 2017.

Women who presented with genito‐urinary or recto‐vaginal fistula following childbirth were eligible for inclusion in this analysis if they had no previous uterine scar and reported that their fistula developed between 1990 and 2014 following a facility delivery. Injuries that could be considered perineal tears were excluded. Women with previous CS are more likely to subsequently deliver by CS; repeat CS was excluded to allow a focus on deliveries following prolonged, obstructed labour. Sixteen women were excluded because of missing information about previous CS. Two women were excluded who did not labour before having CS.

One of the surgeons interviewed the women and recorded information on a standard form,^21^ documenting the woman's sociodemographic data, age at fistula development and obstetric history, including whether labour and delivery that resulted in fistula occurred at home and the sex and condition of the baby. Data were entered into an excel database, with names changed to unique identification numbers to protect the women's privacy. Data were analysed using stata software (StataCorp, 2007; College Station, TX, USA). Approval for this record review was granted by the AMREF Ethics and Scientific Review Committee. There was no patient or public involvement in the analysis.

‘Caesarean section' included CS/hysterectomy and repair of uterine rupture. Vaginal deliveries with missing instrument data were assumed to be spontaneous vaginal deliveries. In cases of multiple gestations, deliveries were counted in the ‘alive' group if at least one baby was alive at birth. Two‐sample *t* tests assume unequal variances. Reported probabilities are associated with Pearson chi‐square tests. Statistical significance was at *P* < 0.05.

## Results

**Figure 1:**
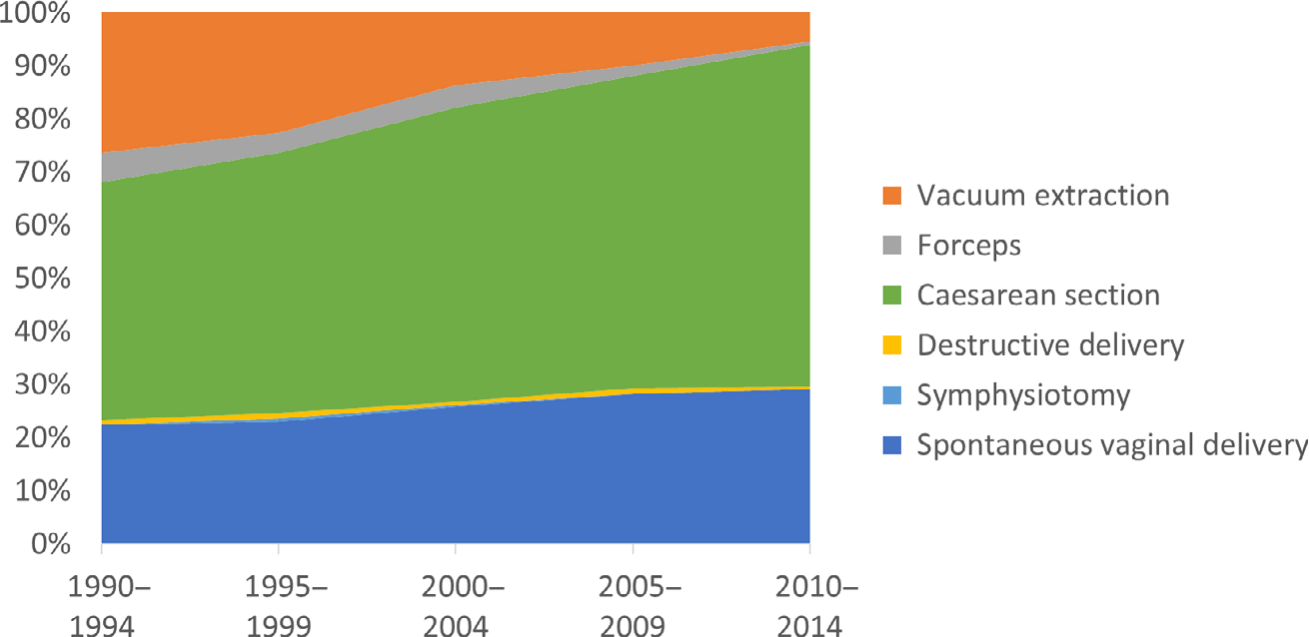
Mode of delivery for prolonged, obstructed labours that resulted in obstetric fistula and stillbirth.

Out of 4396 women who sought fistula treatment following facility delivery, 84.1% had delivered a stillbirth (3695/4396, Table 1). Over half, 57.2% (2515/4396), had undergone CS (Table 2), and stillbirth occurred in 80.7% (2027/2513) of CS deliveries. The frequency of CS with stillbirth increased from 162/361 deliveries (44.9%) in 1990–94 to 331/514 (64.4%) in 2010–14 (*P *< 0.001, Table 3). The analysis includes 63 women operated for uterine rupture repair (1.4% of all women, 2.5% of the 2515 women with CS). Vacuum extraction with stillbirth declined from 95/361 (26.3%) in 1990–94 to 28/514 (5.4%) in 2010–14 (*P *< 0.001, Figure 1). Forceps delivery with stillbirth declined from 20/361 (5.5%) in 1990–94 to 3/514 (0.6%) in 2010–14 (*P *< 0.001).

**Table 1:** Mode of delivery by fetal condition

Mode of delivery	Stillbirth	%	Alive	%	Total
Spontaneous vaginal	955	87.0%	143	13.0%	1098
Vacuum extraction	557	90.6%	58	9.4%	615
Forceps	119	93.0%	9	7.0%	128
Destructive delivery	30	100.0%	0	0.0%	30
Symphysiotomy	7	87.5%	1	12.5%	8
Caesarean section	2027	80.7%	486	19.3%	2513
Total	3695	84.1%	697	15.9%	4392

**Table 2:** Mode of facility delivery for women with genito‐urinary or recto‐vaginal fistula

	1990–94	1995–99	2000–04	2005–09	2010–14	Total
Spontaneous vaginal	95 (22.7%)	219 (22.5%)	348 (24.3%)	254 (27.3%)	184 (28.7%)	1100 (25.0%)
Vacuum extraction	101 (24.2%)	208 (21.4%)	191 (13.3%)	84 (9.0%)	31 (4.8%)	615 (14.0%)
Forceps	22 (5.3%)	33 (3.4%)	54 (3.8%)	15 (1.6%)	4 (0.6%)	128 (2.9%)
Destructive delivery	3 (0.7%)	10 (1.0%)	8 (0.6%)	7 (0.8%)	2 (0.3%)	30 (0.7%)
Symphysiotomy	1 (0.2%)	4 (0.4%)	3 (0.2%)	0 (0%)	0 (0%)	8 (0.2%)
Caesarean section	196 (46.9%)	500 (51.3%)	828 (57.8%)	570 (61.3%)	421 (65.6%)	2515 (57.2%)
Total	418	974	1432	930	642	4396

**Table 3:** Mode of facility delivery for women with genito‐urinary or recto‐vaginal fistula and stillbirth

	1990–94	1995–99	2000–04	2005–09	2010–14	Total
Spontaneous vaginal	81 (22.4%)	195 (23.0%)	310 (25.8%)	219 (28.3%)	150 (29.2%)	955 (25.9%)
Vacuum extraction	95 (26.3%)	192 (22.7%)	165 (13.8%)	77 (9.9%)	28 (5.4%)	557 (15.1%)
Forceps	20 (5.5%)	31 (3.7%)	50 (4.2%)	15 (1.9%)	3 (0.6%)	119 (3.2%)
Destructive delivery	3 (0.8%)	10 (1.2%)	8 (0.7%)	7 (0.9%)	2 (0.4%)	30 (0.8%)
Symphysiotomy	0 (0%)	4 (0.5%)	3 (0.3%)	0 (0%)	0 (0%)	7 (0.2%)
Caesarean section	162 (44.9%)	414 (48.9%)	664 (55.3%)	456 (58.9%)	331 (64.4%)	2027 (54.9%)
Total	361	846	1200	774	514	3695

One‐quarter of women who sought fistula treatment had had a spontaneous vaginal delivery (1100/4396). The proportion of spontaneous vaginal deliveries with stillbirth increased from 81/361 (22.4%) in 1990–94 to 150/514 (29.2%) in 2010–14 (*P *< 0.01). Symphysiotomy and destructive delivery were uncommon. Symphysiotomy accounted for just eight births (0.2%), and seven of those were stillbirths; 30 women (0.7%) reported having a destructive operation for a fetus that had died in utero.

The proportion of stillbirths declined from 361/418 (86.6%) deliveries in 1990–94 to 514/642 (80.2%) in 2010–14 (*P *< 0.01). Early neonatal deaths after CS were 5.6% (141/2513), without significant change over the period.

## Discussion

### Main findings

Over 25 years CS rates rose dramatically in this population of 4396 East and Central African women seeking obstetric fistula repair, even though most babies were stillborn. Increases in CS have been documented in diverse contexts around the world,^11^, ^22^, ^23^ but this analysis is the first to shed light on the frequency of CS with obstructed labour and stillbirth. It indicates widespread disrespect for international guidelines on how to manage obstructed labour with a dead fetus,^24^ illustrating poor quality of care during childbirth.

Why did CS rates with stillbirths increase so dramatically? Governments and health providers are responding to global calls for increasing access to surgical obstetric care in sub‐Saharan Africa.^5^, ^7^, ^25^ All included countries have seen decreases in overall maternal mortality ratios, suggesting that women experiencing obstructed labour increasingly reach facilities. CS is indeed essential: without access to CS some women with obstructed labour would die in spite of all less invasive methods.^22^

Healthcare providers may have misconceptions about the safety of CS, as documented in other populations around the world.^26^, ^27^ Significantly, in many contexts healthcare providers receive more remuneration from performing CS than from attending vaginal births. Although women at risk of obstetric fistula are often unable to pay for services, system‐level financial incentives can motivate clinical decisions, leading to suboptimal care.^28^

Despite clear recommendations in international guidelines for managing women with prolonged, obstructed labour,^24^ the use of vacuum extraction and forceps have declined in diverse global populations.^11^, ^22^, ^23^ Rising CS rates in low‐ and middle‐income countries have not been associated with the maintenance of skills for assisted vaginal delivery, even though this leads to underuse of assisted vaginal delivery in places where CS is least accessible and most unsafe for women.^23^ Vacuum‐assisted deliveries are frequently unavailable in East African facilities categorised as offering basic emergency obstetric care.^4^, ^29^ The biggest drivers of unavailable assisted vaginal delivery have been found to be lack of equipment and lack of trained health providers.^23^

Symphysiotomy could be an option for women who present with obstructed labour and a live baby, as it increases the size of the pelvis to permit vaginal delivery with different short‐ and long‐term risks from CS.^30^, ^31^, ^32^ Most healthcare providers have never benefitted from training, and many dismiss symphysiotomy as an option.^22^, ^30^ Symphysiotomy is not formally encouraged in any country, although it was performed in 8 (0.2%) women.

A small proportion of women with surgical birth had laparotomies to repair uterine rupture (2.5%, 63/2515). Although providers have delivery options when the uterus is intact, there is no alternative mode of delivery to laparotomy for uterine rupture. Uterine rupture may have been suspected in some cases of cephalopelvic disproportion, indicating CS.

It is well recognised that prolonged, obstructed labour is generally fatal to the baby, especially in women who develop fistula.^33^, ^34^ Our data could not capture fetal condition at the time of the CS decision, but 141/2513 (5.6%) women reported early neonatal deaths after CS. Most CS in this series were probably performed when the fetus was already dead, yet destructive procedures were performed in just 30/4396 (0.8%) births in this series, contrary to what is recommended by WHO.^24^

Medical professionals are most comfortable performing procedures that they have learned and practiced. Although some East and Central African countries have stated requirements for interns to conduct a certain number of vaginal deliveries and CS, others do not have clear requirements for licensure. Few countries require experience with vacuum extraction, and significant gaps persist between training ideals and reality on the ground.

Some requirements would be difficult to implement, given that women with obstructed labour and dead babies generally present to remote facilities, far from specialty training in tertiary centres. Nevertheless, medical schools must recognise the realities that many healthcare providers face. Without training emphasis on vacuum extraction and destructive procedures, CS may seem to be the least potentially harmful option, even when not indicated.

The observed decrease in home births over time is a positive development, as good facility care can prevent stillbirths and fistulas. If the quality of care is poor, however, women with prolonged, obstructed labour face an increasing risk that they may deliver by CS and still develop fistula and/or experience stillbirth.

### Strengths and limitations

Although this review analyses a large, multi‐country data set of women with fistula‐related incontinence, it does have limitations. Women with fistulas constitute this cohort, without available comparisons to women who did not develop fistulas. Included women delivered in facilities and later accessed fistula treatment services, which excludes women who delivered at home and women with fistula who did not seek treatment, who might have higher rates of vaginal birth.

This review depends on information from the women about past events. Comparisons between different time periods risk ascertainment bias. Although most women know their obstetric history, in many cases years had passed before they received fistula surgery. Given that we are relying on women's reports, we do not know whether CS was performed during the first or second stage of labour. We do not know the ratio of fresh to macerated stillbirths. We cannot be sure that there was no fetal heartbeat at the time of CS, despite the association between fistula development with prolonged, obstructed labour and intrauterine fetal death.

Our series considers the obstetric history of women who sought repair of genito‐urinary or recto‐vaginal fistula. While most fistulas are obstetric, some fistulas following CS are probably iatrogenic.^21^ Such cases are included in this data set if the woman reported labour, even though some CS leading to iatrogenic fistula may have been performed for unknown indications unrelated to the woman's prolonged, obstructed labour. Inclusion of iatrogenic cases increases the proportion of live births among women reporting CS.

### Interpretation

Healthcare providers must follow evidence‐based guidelines for CS decision‐making, recognising that CS introduces important short‐ and long‐term risks. Training should continue to include CS alternatives, acknowledging that healthcare providers in low‐income settings are often less experienced with instrumental vaginal delivery techniques such as vacuum extraction than with CS. Destructive procedures such as craniotomy allow assisted vaginal delivery of a baby who has died in utero. When indicated, vacuum extraction, forceps, symphysiotomy, or craniotomy can spare mothers from the consequences of unnecessary CS.^19^, ^32^, ^35^ Vacuum extraction should be part of any residency programme in obstetrics. Craniotomy must remain in the training curriculum as long as women arrive at facilities with prolonged, obstructed labour and intrauterine fetal death.

The dramatic rise in CS with stillbirths poses significant risks to women. How should the drivers of this harmful care be addressed?^28^, ^36^, ^37^ Careful reconsideration of provider payments and incentives could reduce financial motivations that favour unnecessary CS. Elsewhere in the world, patient advocacy and threat of litigation often reinforce provider commitment to evidence‐based, patient‐centred care. Although women with fistulas are often marginalised and disempowered, informed women could demand improvements, including in remote, under‐resourced facilities.^38^

## Conclusion

In Eastern and Central Africa, CS is increasingly performed on women with obstructed labour whose babies have already died in utero. Contrary to international recommendations, alternatives such as vacuum extraction and destructive delivery are decreasingly used. Training programmes, policies and protocols must enable providers to overcome challenges related to knowledge, bias and uncertainty. Women who develop fistula and stillbirth following prolonged, obstructed labour must be spared the consequences of unnecessary CS.

### Disclosure of interests

None. Completed disclosure of interests forms are available to view online as supporting information.

### Contribution to authorship

CJN and TJIPR designed the study, interpreted the data, and wrote the manuscript. TJIPR collected data. CJN developed data coding, analysed data, and developed tables and figure. LL conducted literature searches and entered data. JR, SW and MT interpreted data and provided reviews.

### Details of ethics approval

AMREF Ethics and Scientific Review Committee P88/2013, 17 February 2014, renewed 20 November 2018.

### Funding

None.

### Acknowledgements

We sincerely thank Dr Marietta Mahendeka for her collaboration repairing fistulas and collecting data. We thank the African Medical and Research Foundation (AMREF), EngenderHealth, the Fistula Foundation, the Freedom from Fistula Foundation, Gesellschaft für Technische Zusammenarbeit (GTZ; now GIZ), Johnson & Johnson, the Royal Netherlands Embassy of Tanzania, SOS East Africa, United Nations Population Fund (UNFPA), and Women and Health Alliance International (WAHA) for supporting the second author in travelling to the many hospitals. We thank Millicent Oundo for entering some of the data through the support of the United States Agency for International Development through EngenderHealth's Fistula Care project. We are grateful to the Fistula Foundation and the International Federation of Gynecology and Obstetrics (FIGO) for paying the BJOG open access fee. We thank the specialists and staff in the hospitals, who operated on the women and managed them during their hospital stays. Finally, we thank the women whose unforgettable experiences inform our conclusions.

## Supporting information


**Table S1**. Country where women sought fistula treatment.

## References

[1] McClure EM , Goldenberg RL , Bann CM . Maternal mortality, stillbirth and measures of obstetric care in developing and developed countries. Int J Gynecol Obstet 2007;96:139–46. 10.1016/j.ijgo.2006.10.010 17274999

[2] Lawn JE , Blencowe H , Pattinson R , Cousens S , Kumar R , Ibiebele I , et al. Stillbirths: Where? When? Why? How to make the data count? Lancet 2011;377:1448–63. 21496911 10.1016/S0140-6736(10)62187-3

[3] Campbell OMR , Graham WJ . Strategies for reducing maternal mortality: getting on with what works. Lancet 2006;368:1284–99. 17027735 10.1016/S0140-6736(06)69381-1

[4] Pearson L , Shoo R . Availability and use of emergency obstetric services: Kenya, Rwanda, Southern Sudan, and Uganda. Int J Gynecol Obstet 2005;88:208–15. 10.1016/j.ijgo.2004.09.027 15694109

[5] Betrán AP , Merialdi M , Lauer JA , Bing‐Shun W , Thomas J , Van Look P , et al. Rates of caesarean section: analysis of global, regional and national estimates. Paediatr Perinat Epidemiol 2007;21:98–113. 17302638 10.1111/j.1365-3016.2007.00786.x

[6] Ye J , Zhang J , Mikolajczyk R , Torloni MR , Gülmezoglu AM , Betran AP . Association between rates of caesarean section and maternal and neonatal mortality in the 21st century: a worldwide population‐based ecological study with longitudinal data. BJOG 2016;123:745–53. 26331389 10.1111/1471-0528.13592 PMC5014131

[7] Harrison MS , Pasha O , Saleem S , Ali S , Chomba E , Carlo WA , et al. A prospective study of maternal, fetal and neonatal outcomes in the setting of cesarean section in low‐ and middle‐income countries. Acta Obstet Gynecol Scand 2017;96:410–20. 28107771 10.1111/aogs.13098 PMC5665564

[8] Betran AP , Torloni MR , Zhang JJ , Gülmezoglu AM , WHO Working Group on Caesarean Section . WHO Statement on Caesarean Section Rates. BJOG 2016;123:667–70. 26681211 10.1111/1471-0528.13526 PMC5034743

[9] Silver RM . Implications of the first cesarean: perinatal and future reproductive health and subsequent cesareans, placentation issues, uterine rupture risk, morbidity, and mortality. Semin Perinatol 2012;36:315–23. 23009962 10.1053/j.semperi.2012.04.013

[10] Motomura K , Ganchimeg T , Nagata C , Ota E , Vogel JP , Betran AP , et al. Incidence and outcomes of uterine rupture among women with prior caesarean section: WHO Multicountry Survey on Maternal and Newborn Health. Sci Rep 2017;10:44093. 10.1038/srep44093 PMC5345021 28281576

[11] Dekker L , Houtzager T , Kilume O , Horogo J , van Roosmalen J , Nyamtema AS . Caesarean section audit to improve quality of care in a rural referral hospital in Tanzania. BMC Pregnancy Childbirth 2018;18:164. 29764384 10.1186/s12884-018-1814-1 PMC5952645

[12] Sandall J , Tribe RM , Avery L , Mola G , Visser GH , Homer CS , et al. Short‐term and long‐term effects of caesarean section on the health of women and children. Lancet 2018;392:1349–57. 30322585 10.1016/S0140-6736(18)31930-5

[13] Bishop D , Dyer RA , Maswime S , Rodseth RN , van Dyk D , Kluyts H‐L , et al. Maternal and neonatal outcomes after caesarean delivery in the African Surgical Outcomes Study: a 7‐day prospective observational cohort study. Lancet Glob Health 2019;7:e513–22. 30879511 10.1016/S2214-109X(19)30036-1

[14] Sobhy S , Arroyo‐Manzano D , Murugesu N , Karthikeyan G , Kumar V , Kaur I , et al. Maternal and perinatal mortality and complications associated with caesarean section in low‐income and middle‐income countries: a systematic review and meta‐analysis. Lancet 2019;393:1973–82. 30929893 10.1016/S0140-6736(18)32386-9

[15] Souza JP , Gülmezoglu A , Lumbiganon P , Laopaiboon M , Carroli G , Fawole B , et al. Caesarean section without medical indications is associated with an increased risk of adverse short‐term maternal outcomes: the 2004–2008 WHO Global Survey on Maternal and Perinatal Health. BMC Med 2010;10:71. 10.1186/1741-7015-8-71 PMC2993644 21067593

[16] Belizán JM , Althabe F , Cafferata ML . Health consequences of the increasing caesarean section rates. Epidemiology 2007;18:485–6. 17568221 10.1097/EDE.0b013e318068646a

[17] WHO, Unicef, others . AMDD. Monitoring Emergency Obstetric Care: A Handbook. Geneva: WHO; 2009:152.

[18] Hardee K , Gay J , Blanc AK . Maternal morbidity: neglected dimension of safe motherhood in the developing world. Glob Public Health 2012;7:603–17. 22424546 10.1080/17441692.2012.668919 PMC3396379

[19] Khisa AM , Nyamongo IK . Still living with fistula: an exploratory study of the experience of women with obstetric fistula following corrective surgery in West Pokot, Kenya. Reprod Health Matters 2012;20:59–66. 23245409 10.1016/S0968-8080(12)40661-9

[20] Zheng AX , Anderson F . Obstetric fistula in low‐income countries. Int J Gynecol [Internet]. 2009;104:85–89. 10.1016/j.ijgo.2008.09.011 19027903

[21] Raassen TJIP , Ngongo CJ , Mahendeka MM . Iatrogenic genitourinary fistula: an 18‐year retrospective review of 805 injuries. Int Urogynecol J 2014;25:1699–706. 25062654 10.1007/s00192-014-2445-3 PMC4234894

[22] Hofmeyr GJ . Obstructed labor: using better technologies to reduce mortality. Int J Gynecol Obstet 2004;85(Suppl 1):S62–72. 10.1016/j.ijgo.2004.01.011 15147855

[23] Bailey PE , van Roosmalen J , Mola G , Evans C , de Bernis L , Dao B . Assisted vaginal delivery in low and middle income countries: an overview. BJOG 2017;124:1335–44. 28139878 10.1111/1471-0528.14477

[24] World Health Organization, UNICEF, United Nations Population Fund . Managing Complications in Pregnancy and Childbirth: A Guide for Midwives and Doctors, 2nd edn. Geneva: World Health Organization, editor. 2017.

[25] Irani M , Deering S . Challenges affecting access to cesarean delivery and strategies to overcome them in low‐income countries. Int J Gynecol Obstet 2015;131:30–4. 10.1016/j.ijgo.2015.04.036 26115791

[26] Betrán AP , Ye J , Moller A‐B , Zhang J , Gülmezoglu AM , Torloni MR . The increasing trend in caesarean section rates: Global, regional and national estimates: 1990–2014. PLoS One 2016;11:e0148343. 26849801 10.1371/journal.pone.0148343 PMC4743929

[27] D'Souza R . Caesarean section on maternal request for non‐medical reasons: putting the UK National Institute of Health and Clinical Excellence guidelines in perspective. Best Pract Res Clin Obstet Gynaecol 2013;27:165–77. 23116717 10.1016/j.bpobgyn.2012.09.006

[28] Saini V , Garcia‐Armesto S , Klemperer D , Paris V , Elshaug AG , Brownlee S , et al. Drivers of poor medical care. Lancet 2017;390:178–90. 28077235 10.1016/S0140-6736(16)30947-3

[29] Bailey PE . The disappearing art of instrumental delivery: time to reverse the trend. Int J Gynaecol Obstet 2005;91:89–96. 16109417 10.1016/j.ijgo.2005.05.016

[30] Verkuyl DAA . Think globally act locally: the case for symphysiotomy. PLoS Med 2007;4:e71. 17388656 10.1371/journal.pmed.0040071 PMC1831724

[31] Monjok E , Okokon IB , Opiah MM , Ingwu JA , Ekabua JE , Essien EJ . Obstructed labour in resource‐poor settings: the need for revival of symphysiotomy in Nigeria. Afr J Reprod Health 2012;16:94–101. 23437503

[32] Maharaj D , Moodley J . Symphysiotomy and fetal destructive operations. Best Pract Res Clin Obstet Gynaecol 2002;16:117–31. 11866501 10.1053/beog.2001.0259

[33] Cowgill KD , Bishop J , Norgaard AK , Rubens CE , Gravett MG . Obstetric fistula in low‐resource countries: an under‐valued and under‐studied problem – systematic review of its incidence, prevalence, and association with stillbirth. BMC Pregnancy Childbirth 2015;26:193. 10.1186/s12884-015-0592-2 PMC4550077 26306705

[34] Goldenberg RL , McClure EM , Bhutta ZA , Belizán JM , Reddy UM , Rubens CE , et al. Stillbirths: the vision for 2020. Lancet 2011;377:1798–805. 21496912 10.1016/S0140-6736(10)62235-0

[35] Litorp H , Kidanto HL , Nystrom L , Darj E , Essén B . Increasing caesarean section rates among low‐risk groups: a panel study classifying deliveries according to Robson at a university hospital in Tanzania. BMC Pregnancy Childbirth 2013;8:107. 10.1186/1471-2393-13-107 PMC3655870 23656693

[36] Kleinert S , Horton R . From universal health coverage to right care for health. Lancet 2017;390:101–2. 28077231 10.1016/S0140-6736(16)32588-0

[37] Betrán AP , Temmerman M , Kingdon C , Mohiddin A , Opiyo N , Torloni MR , et al. Interventions to reduce unnecessary caesarean sections in healthy women and babies. Lancet 2018;392:1358–68. 30322586 10.1016/S0140-6736(18)31927-5

[38] Nolens B , van den Akker T , Lule J , Twinomuhangi S , van Roosmalen J , Byamugisha J . Women's recommendations: vacuum extraction or caesarean section for prolonged second stage of labour, a prospective cohort study in Uganda. Trop Med Int Health 2019;25:553–62. 10.1111/tmi.13222 PMC6850599 30803113

