# Supplementary material for: Delivery mode for prolonged, obstructed labour resulting in obstetric fistula: a retrospective review of 4396 women in East and Central Africa
Source: BJOG. 2020 Jan 2;127(6):702–7. doi: 10.1111/1471-0528.16047 (PMC7187175; doi:10.1111/1471-0528.16047)
Supplement: Supplementary file 1 — Table S1. Country where women sought fistula treatment. [file BJO-127-702-s001.pdf]

Table S1. Country where women sought fistula treatment

Country	Frequency	Percent of sample
Tanzania	1,587	36.1
Uganda	994	22.6
Kenya	678	15.4
Malawi	428	9.7
Rwanda	297	6.8
Somalia	145	3.3
South Sudan	98	2.2
Zambia	113	2.6
Ethiopia	56	1.3
**Total**	**4,396**	**100.0**
